# Beyond ventilator weaning: rethinking the role of tracheostomy in Guillain-Barré syndrome

**DOI:** 10.1016/j.aicoj.2026.100155

**Published:** 2026-09-14

**Authors:** Clémence Marois, François Beloncle, Djillali Annane

**Affiliations:** a Service de Médecine Intensive Réanimation Neurologique, DMU Neurosciences, AP-HP, Groupe Hospitalier Universitaire APHP-Sorbonne Université, Site Pitié-Salpêtrière, Paris, France; b Groupe de Recherche Clinique en REanimation et Soins intensifs du Patient en Insuffisance Respiratoire aiguE (GRC-RESPIRE) Sorbonne Université, Paris, France; c Medical Intensive Care Unit, CHU Angers, Univ Angers, Vent’Lab, CARE, SFR ICAT, F-49000, Angers, France; d Service de Médecine Intensive Réanimation, DMU Neurolocomoteur et Handicaps, Groupe Hospitalier Universitaire AP-HP Université Paris Saclay, Site Raymond Poincaré, Garches, France; e Centre de Référence des Maladies Rares neuromusculaires (CRMR) Nord – Est – Ile-de-France (NEIDF), Filière neuromusculaire (FILNEMUS), Hôpital Raymond Poincaré, Garches, France; f IHU SEPSIS, PROMETHEUS Comprehensive Sepsis Centre, Université de Versailles Saint Quentin (UVSQ), Université Paris Saclay (UPSaclay), CEA, INSERM, APHP, Hôpital Raymond Poincaré, Garches, France

Dear Editor-in-chief,

Guillain-Barré syndrome (GBS) is a major neurological cause of acute respiratory failure requiring prolonged mechanical ventilation in the intensive care unit (ICU). Approximately one quarter of patients require invasive mechanical ventilation, often for several weeks, while awaiting recovery of bulbar and respiratory muscle function [1]. During this period, clinicians are frequently confronted with a difficult decision: should tracheostomy be performed directly before spontaneous breathing trials (SBT), or extubation attempt, or only after an initial standard weaning strategy has failed?

In this issue of Annals of Intensive Care, Thille and colleagues [2] address this important and understudied clinical question in a large French nationwide multicentre cohort. Their retrospective cohort included 494 patients intubated for GBS between 2014 and 2023. At day 28 after weaning initiation, 43% of patients in the direct tracheostomy group and 83% of those in the standard weaning group were alive and free from mechanical ventilation. G-computation yielded an estimated marginal risk difference of −39.5% (95% CI −47.3 to −31.5 percentage points) for direct versus standard weaning. Direct tracheostomy was associated with a substantially lower probability of being alive and free from mechanical ventilation at day 28. These findings challenge the assumption that bypassing an initial weaning attempt benefits patients expected to require prolonged ventilatory support.

However, beyond the comparison of two strategies, this study raises a more fundamental question: are we evaluating tracheostomy using the appropriate outcomes in patients with neuromuscular respiratory failure? Unlike most critically ill patients, in GBS, liberation from mechanical ventilation is determined largely by neurological recovery rather than resolution of primary lung injury, including restoration of inspiratory and expiratory muscles strength and function, effective cough and secretion clearance, airway protection and swallowing function. Consequently, mechanical ventilation duration is strongly influenced by the neurological recovery trajectory, although the airway and weaning strategy may also contribute. This distinction broadens the objectives against which tracheostomy should be evaluated.

In general ICU populations, trials of tracheostomy timing have primarily evaluated mortality, ventilator associated pneumonia, mechanical ventilation duration, and ICU length of stay [3,4]. Although these outcomes remain clinically relevant, they may not fully capture the potential value of tracheostomy in patients with prolonged neuromuscular respiratory failure. In GBS, tracheostomy is generally considered when prolonged mechanical ventilation is anticipated [5]. The performance of tracheostomy is therefore both a marker of anticipated disease severity and an intervention that may itself influence sedation, mobilization, airway management, and weaning. The decision relies on a comprehensive bedside assessment integrating multiple clinical features including the disease phase, limb and respiratory muscles strength, bulbar dysfunction, cough effectiveness, dysautonomia, electrophysiological evidence of axonal degeneration, and tolerance of assisted spontaneous ventilation. Not all these time-varying features can be fully captured in retrospective databases.

The interpretation of observational comparisons between direct tracheostomy and standard weaning strategies remains challenging. The authors appropriately used g-computation to estimate a marginal causal effect and adjust for measured baseline differences. Nevertheless, residual confounding remains possible because treatment allocation in GBS depended on complex, partly time-varying clinical assessments. The decision to perform a tracheostomy before SBT failure may reflect clinicians’ anticipation of delayed neurological recovery and overall disease severity. Consequently, the estimated effect may combine a true effect of the strategy with residual confounding by indication.

A second issue concerns the definition and alignment of time zero for weaning. In GBS patients who underwent direct tracheostomy, the procedure may not represent the physiological onset of the weaning, because some patients remain fully dependent on controlled ventilation. Using tracheostomy as time zero in one group and the first SBT or extubation attempt in the other may compare patients at different stages of neurological recovery. Retrospective ascertainment of SBTs is also difficult, and criteria for initiating an SBT were not standardized. Clinician-dependent neurological assessment may therefore have affected both group assignment and the timing of outcome measurement.

Beyond its possible effect on ventilation duration, tracheostomy may have other patient-centered objectives in GBS. Potential benefits include lower sedation requirements, improved comfort and communication, easier secretion management, and earlier rehabilitation; these must be balanced against procedural complications, airway injury, infection, and delayed decannulation [5,6]. These outcomes were not assessed by Thille et al. and therefore cannot counterbalance or explain the observed difference in ventilator liberation. Future studies should include sedation exposure, comfort and communication, swallowing and airway protection, rehabilitation milestones, functional recovery, time to decannulation, quality of life, and long-term neurological outcomes.

In GBS, successful respiratory management cannot be judged solely by the earliest possible liberation from mechanical ventilation. The findings of Thille et al. do not support routine direct tracheostomy before an initial weaning assessment; however, they do not establish that tracheostomy lacks value for carefully selected patients expected to undergo prolonged neurological recovery. The next step is to determine which patients benefit from tracheostomy, when it should be performed, and which outcomes best capture its overall value.

## Consent for publication

Not applicable.

## Ethics approval and consent to participate

Not applicable.

## Declaration of Generative AI and AI-assisted technologies in the writing process

ChatGPT was used to assist with improving the English language of the editorial. The authors remain fully responsible for the content and final wording of the manuscript.

## Funding

Not applicable.

## Declaration of competing interest

CM received consultant fees from Alexion and Johnson & Johnson, and payment for presentation from ArgenX, outside the present work.

DA declare to be member of Myasthénie Gravis Scientific advisory board Alexion- Astra Zeneca and Principal Investigator CHAMPION and PREVAIL trial on anti C5 for Myasthenia Gravis outside the present work.

FB have no competing interest.

The authors declare that they have no known competing financial interests or personal relationships that could have appeared to influence the work reported in this paper.
